# Supplementary figures and images for: Targeted Whole Genome Sequencing (TWG-Seq) of Cucumber Green Mottle Mosaic Virus Using Tiled Amplicon Multiplex PCR and Nanopore Sequencing
Source: Plants (Basel). 2022 Oct 14;11(20):2716. doi: 10.3390/plants11202716 (PMC9607580; doi:10.3390/plants11202716)

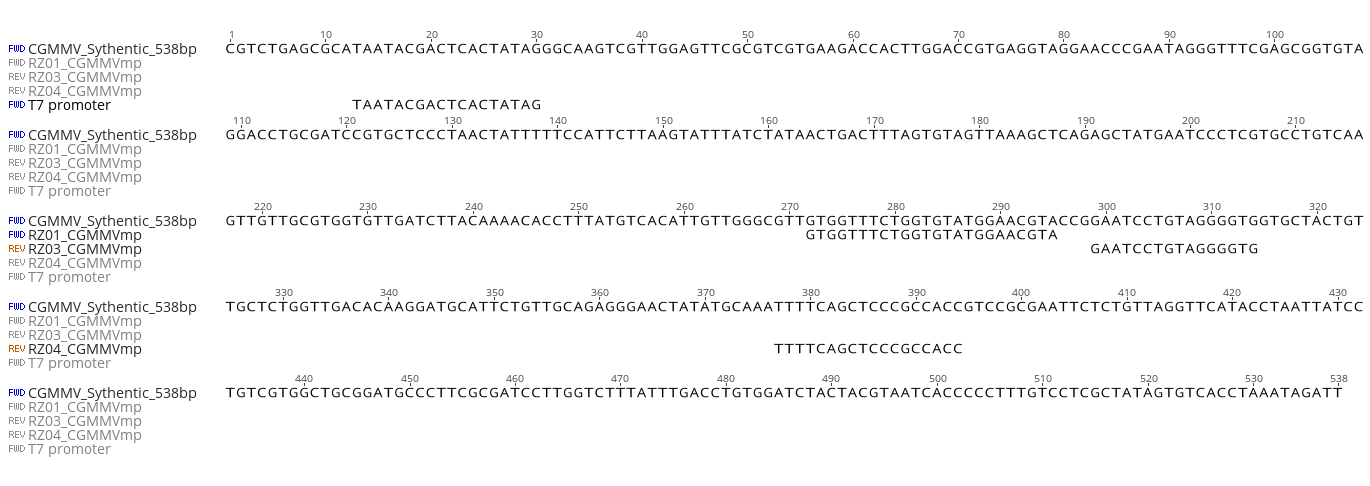

Supplement: Supplementary file 1 [file plants-11-02716-s001.zip › Figure S1.jpg]

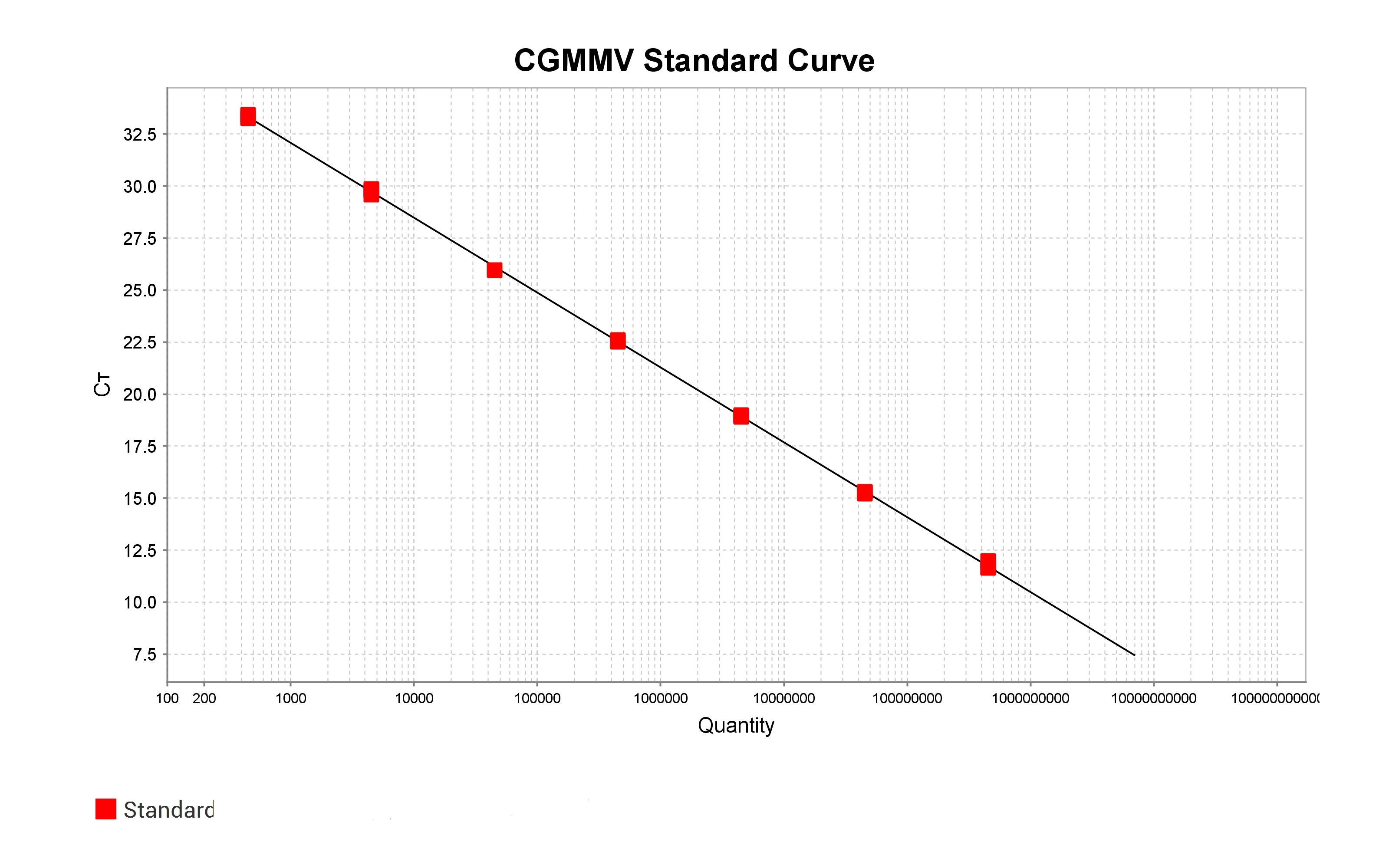

Supplement: Supplementary file 1 [file plants-11-02716-s001.zip › Figure S2.jpg]
